# Effect of high-voltage electrical stimulation on the albumin and histamine serum concentrations, edema, and pain in acute joint inflammation of rats

**DOI:** 10.1590/bjpt-rbf.2014.0079

**Published:** 2015-04-27

**Authors:** Maria C. Sandoval, Carolina R. Ramirez, Diana M. Camargo, Thiago L. Russo, Tania F. Salvini

**Affiliations:** 1 Escola de Fisioterapia, Universidade Industrial de Santander (UIS), Bucaramanga, Colômbia; 2 Departamento de Fisioterapia, Universidade Federal de São Carlos (UFSCar), São Carlos, SP, Brazil

**Keywords:** physical therapy, electrical stimulation, sprain, inflammation

## Abstract

**BACKGROUND:**

The mechanism by which high-voltage electrical stimulation (HVPC) acts on edema reduction is unknown.

**OBJECTIVE:**

To assess the effect of HVPC with negative polarity (-) applied to the ankle of rats with acute joint inflammation.

**METHOD:**

Sixty-four rats were divided into four groups (n=16): inflamed+HVPC(-), 0.03 mL application of ι-carrageenan (3%) to the tibiotarsal joint plus HVPC(-); inflamed+HVPC placebo, carrageenan application and HVPC placebo; normal+HVPC(-), HVPC application(-); and normal control, no intervention. The HVPC(-) 100 Hz at a submotor level was applied daily for 45 min on three consecutive days. The variables were pain, hind-foot volume, and serum histamine and albumin assessed before and during the 48 hours following inflammation. The variables were compared using the *t* test, one-way ANOVA, nested ANOVA for repeated measures, and the *post hoc* Bonferroni test. Analysis of covariance was applied to adjust the effects of HVPC(-) by measurements of pain, inflammation, albumin, and histamine at 24 h, and the final weight was compared to the other groups. The significance level was set at p<0.05.

**RESULTS:**

There were no differences between the inflamed+HVPC(-) and inflamed+HVPC placebo groups in terms of pain or edema (p>0.05). Albumin was reduced in the groups that received the intervention, but there was no differences between them. There was only a 24 hour increase in histamine with the normal+HVPC(-) (p=0.0001) and inflamed+HVPC placebo groups (p=0.01) compared to the normal control group.

**CONCLUSIONS:**

The results of the present study suggest that HVPC(-) with the parameters employed did not reduce pain or edema and did not change serum albumin or histamine levels,, which indicates the inability of this resource to have a positive effect when treating treat acute joint inflammation.

## Introduction

The individual's ability to return to normal activity in the presence of joint inflammation often depends on the reduction of pain and edema^1^^,^ 2. Therefore, the control of pain and edema are essential for a quick recovery of musculoskeletal function.

High-voltage electrical stimulation (high-voltage pulsed current - HVPC) is one of the treatments used in physical therapy to control these symptoms. HVPC is a monophasic pulsed electric current that consists of double-peaked impulses with a very short duration (5-100 μs) and longer interpulse intervals, which generates a low total current (1.5 mA), despite a voltage greater than 150 V. It has been reported that in animals, the use of the monophasic current^3^ with negative polarity (-) and a submotor level of stimulation^4^^-^ 8 reduces acute post-traumatic edema^3^^,^ 4. Other studies using low-voltage current^9^ with positive polarity^10^ and motor-level stimulation^11^ have shown no reduction in edema. To our knowledge, only one study has demonstrated clinical differences in the reduction of chronic post-traumatic hand edema in humans^1^.

The mechanisms by which HVPC(-) acts to decrease edema have not been extensively studied, and several hypotheses have been proposed. One of these hypotheses is the electrophysiological phenomenon described by Cosgrove et al.^12^ that assumes that the HVPC(-) repels the proteins, causing their displacement in the interstitium of the traumatized area, accelerating protein uptake by the lymphatic capillaries, which, in turn, facilitates lymphatic flow and decreases the interstitial oncotic pressure and edema. This hypothesis was partially supported by Reed^13^, who reported that HVPC(-) at 30 and 50 V significantly decreased the permeability to the macromolecules, and then limited the edema formation, particularly in the acute phase. Another hypothesis suggested that HVPC(-) stimulated lymphatic flow and then facilitated its drainage^5^^,^ 14. Based on the hypothesis of Cosgrove et al.^12^, Cook et al.^15^ suggested that HVPC(-) facilitated albumin transport, increasing its movement, which then increased the diameter of lymphatic lumen and caused contraction of lymphatic smooth muscle. Karnes et al.^9^ proposed other hypotheses: 1) HVPC(-) contracted the smooth muscle surrounding blood vessels, decreasing the diameter of arterioles; 2) because HVPC(-) is polarized, it generated the accumulation of electric charges, which then changed the pH and decreased the edema.

Taylor et al.^3^ also reported that HVPC(-) reduced the diameter of arterioles and thereby reduced the blood flow, which might be another mechanism of action. Therefore, HVPC(-) could either influence histamine release from cells or affect the histamine-binding site of the histamine receptors in the postcapillary-venule endothelial cells. These hypotheses hold that albumin and histamine were important for the positive effects of HVPC(-). Albumin accounts for 50% of total plasma protein and is responsible for 75% of the colloid osmotic pressure^16^. Histamine is a dibasic vasoactive amine secreted at the beginning of the inflammation process. It causes arteriolar dilation, increasing the vascular permeability of the large venules, which then allows the movement of fluid and proteins into the extravascular space^17^. Therefore, albumin and histamine are important inflammation markers.

Based on this background, the hypothesis of the present study was that HVPC(-) decreased edema and the production of histamine and reduced the movement of albumin into the interstitial space, as a result of reduced endothelial permeability. The present study aimed to assess the effect HVPC(-) applied to the rat ankle on pain, edema, and serum levels of albumin and histamine in acute joint inflammation.

## Method

### Animals

Sixty-four male Wistar rats (311.5±36.1 g) were kept in plastic cages (four animals per cage), with water and pelleted food *ad libitum*. They were maintained in a controlled environment with a 12:12 h light-dark cycle and temperature of 22±2°C. At the end of the experimental protocol (48 hours), the animals were killed by anesthetic overdose. This study was approved by the Animal Experimentation Ethics Committee of the *Universidade Federal de São Carlos* (UFSCar), São Carlos, SP, Brazil (No. 027/2007).

### Experimental groups

The animals were randomly divided into four groups (16 animals per group).

**Inflamed+HVPC(-) group:** animals received an injection of ι-carrageenan in the ankle of the right paw, followed by immediate application of HVPC(-).

**Inflamed+HVPC Sham group:** animals received an injection of ι-carrageenan, followed by the placement of the HVPC electrodes, but without the application of the current.

**Normal+HVPC(-) group:** animals were submitted to HVPC(-) with the same parameters used in the intervention group.

**Normal control group (NCG):** this group received no intervention.

The animals were weighed before and after the experiment. For all experimental procedures and euthanasia, animals were anesthetized through intraperitoneal injection of a cocktail containing xylazine (12 mg/kg) and ketamine (95 mg/kg). The dorsal region of the trunk and both hind legs were shaved to visualize the sites of carrageenan injection and electrode placement.

### Joint inflammation procedure

The inflammation was induced in the right tibiotarsal joint by an injection of 0.03 mL of 3% ι-carrageenan (Sigma Chemical Company, St. Louis, USA) dissolved in saline solution (0.9% NaCl). The administration followed the method described by Omote et al.^18^. All joint inflammation was induced in the morning.

**Volume assessment:** Volumetry was used to assess the volume, which is considered a gold-standard method with high reproducibility (intraclass correlation coefficient (ICC)=0.99) and an error less than 1%^19^. A glass cup constructed especially to fit the animal paw (5 cm high and 4 cm in diameter) was used. It had been previously evaluated to ensure reproducibility in the volume measurements (ICC=0.95). For the measurements, the lateral region of the rat's ankle was marked, measuring 1 cm from the base of the heel. Following the ankle marking, the animal was suspended in a sling similar to that used by Dolan et al.^6^^,^ 7 in their studies. Water was placed in the cup to the maximum level, allowing the liquid leakage to stabilize. Then, the paw was immersed up to the level of the paw's ink mark. The displaced water was collected and measured, where 1 mL = 1 mg. This measurement was performed before the application of carrageenan, as well as 5, 24, and 48 hours after the induction of inflammation.

**Pain evaluation:** A paw withdrawal latency to radiant heat was used^20^^-^ 23, which is a method with high reproducibility (r2=0.78)23. The animal was placed in a glass box (20 cm long, 15 cm wide, 15 cm high), which was maintained on an elevated platform with a lamp under the right paw. After 10 minutes of acclimation, the lamp and the timer were simultaneously turned on, and the time at which the animal withdrew its paw from the bottom of the box was recorded (in seconds); this interval was noted as the paw withdrawal latency (PWL)^20^. This assessment was repeated three times with 5 minutes of rest between each measurement. The median of the values obtained in the three assessments was calculated. The PWL was assessed 12 hours before starting the experiment and 6, 24, and 48 hours after the induction of inflammation. Pain was assessed 1 hour after measuring paw volume because the animal needed to be awake for the test.

**Serum levels of albumin and histamine:** Two milliliters of blood was collected from the animals of the electrically stimulated and placebo groups 24 and 48 hours after injection of carrageenan. Only one collection was performed in the NCG group. Blood was collected from 8 of the 16 animals in each group. The first blood sample was collected from the tail vein, and the second sample was collected from the renal artery. Albumin was processed using the bromocresol green method^24^ and quantified in a spectrophotometer at a wavelength of 630 nm. Histamine was measured by radioimmunoassay^24^.

Volume and pain assessment and blood collection were performed between 8 am and 2 pm, according to the time that inflammation was induced in each animal.

### HVPC protocol

HVPC was applied with a Neurodyn High Volt (Ibramed, Brazil), with current characterized by double-peak monophasic pulses. The HVPC protocol was as follows: pulse duration of 20 μs, interpulse interval of 100 μs, frequency of 100 pps, and amplitude at a submotor level. The pressure was gradually increased until a visible twitch was observed, and then it was decreased to a threshold immediately below the muscle contraction. Negative polarity was used with two square, active, self-adhesive electrodes (1 cm^2^), attached with adhesive tape on the lateral and medial regions of the tibiotarsal joint^1^. A rectangular dispersive self-adhesive electrode (9x3 cm) was maintained in the dorsal region of the animal's trunk. During the application of current, the animals were kept in a warm environment to prevent hypothermia caused by anesthesia. The inflamed+HVPC(-) group and the inflamed+HVPC Sham group were submitted to the HVPC treatment immediately after the carrageenan injection, while the normal+HVPC(-) group was submitted to this treatment after shaving. All animals received a daily session of electrical stimulation or Sham for 45 minutes on three consecutive days.

### Statistical analysis

The paired student's t-test was used to determine possible differences (i.e. initial vs. final) in weight, histamine, and albumin between groups of animals; the percentage of weight variation was also determined [i.e. (final mass x 100)/initial mass]. One-way analysis of variance (ANOVA) was used to describe and compare the variables between the groups, and the post hoc Bonferroni test was applied when differences were observed. The Kruskal-Wallis test was used to determine the differences in the variable pain between groups. Repeated-measures ANOVA (i.e. nested ANOVA) was used to analyze the volume and pain values obtained at different times. In addition, analysis of covariance was used for the output variables (i.e. pain, inflammation, histamine, and albumin) 48 hours after the induction of inflammation to evaluate the differences between the inflamed+HVPC(-) group and the other groups. With this analysis, the results were adjusted by the measurements performed at 0, 5, 6, and 24 hours, as well as by the initial and final weight of the animals. The data were processed with STATA 9.0 software. The significance level was set at p<0.05.

## Results

### Body weight

In all groups, there was a difference between the initial and the final weight. The three groups that received intervention presented weight loss, which was greater in the inflamed+HVPC(-) group (Table 1).

**Table 1.** Comparison between initial and final mass within each group of mice (mean±SD).

	Normal Control	Normal+ HVPC(-)	Inflamed+ HVPC Sham	Inflamed + HVPC(-)
Initial mass (g)	283.4±34.2	327.1±35.3	324.6±32.9	310.9±26.4
Final mass (g)	291.9±35.6	316.1±31.7	308.2±30.8	289.6±23*
p (initial vs. final)	<0.0001	<0.0001	<0.0001	<0.0001
% variation	+3%	3.38%	5.05%	6.9%

* p<0.05 compared to normal+ HVPC(-) % variation: [ (final mass x 100)/initial mass)]. HVPC: High voltage electrical stimulation; p: statistical significance

### Pain

No significant differences were observed in PWL between groups at any time-point (Table 2). Similarly, no significant differences were observed in the PWL between the beginning and end of the experiment in any group (Table 2). Additionally, there were no differences by treatment or by time-point (p=0.09 and p=0.33, respectively).

**Table 2.** Paw withdrawal latency time over the course of 48 hours within each group of mice.

Test Time	Normal Control	Normal + HVPC(-)	Inflamed + HVPC Sham	Inflamed + HVPC(-)	p (between groups)
0	26.5 [19-40]s	37.5 [18-64]s	25.5 [16-34]s	31.5 [24.5-51]s	0.25
6			24.5 [17.5-49.5]s	29 [17-58]s	0.83
24			28.5 [18-49]s	32.5 [20.5-50.5]s	0.45
48		31 [21-51]s	24.5 [15.5-32]s	32.5 [21.5-58.5]s	0.36
p (0 vs. 48 h)		0.80	0.89	0.85	

Data are presented as median ± [Standard Deviation]. HVPC: High voltage electrical stimulation; S: seconds. P: statistical significance.

### Volume

All groups showed similar paw volume at baseline (p=0.65). There was a significant increase in paw volume 24 hours after the induction of inflammation compared with baseline in the inflamed+HVPC(-) and inflamed+HVPC placebo groups, and this difference was also observed at 48 hours. However, there was no difference between these two groups in any of the analyzed time-points. There were no differences by treatment (p=0.22) or by time-point (p=0.057).

### Albumin

There was no difference in serum albumin at 24 hours between the groups (p=0.34). Serum albumin was lower at 24 than at 48 hours in the normal+HVPC(-), inflamed+HVPC placebo, and inflamed+HVPC(-) groups, with no difference between these groups (p=0.89; Table 3).

**Table 3.** Comparison of albumin (g/dL) between 24 and 48 hours within each group of mice.

Time	Normal Control	Normal + HVPC(-)	Inflamed + HVPC Sham	Inflamed+ HVPC(-)

24 h	2.52±0.54	2.76±0.24	2.83±0.37	2.8±0.3
48 h		2.46±0.29*	2.34±0.2*	2.3±0.33*
p (24 vs. 48 h)		0.03	<0.0001	0.004

Data are expressed as the mean ± SD.

* p<0.05 compared to their respective values at 24 hours. HVPC: High voltage electrical stimulation. P: statistical significance

### Histamine

Serum histamine was different between the groups at 24 hours (p=0.006; Table 4). However, only the normal+HVPC(-) and inflamed+HVPC placebo groups presented a significant increase in histamine compared with NCG (p=0.001 and p=0.01, respectively). At 48 hours, a reduction in histamine was observed in the three groups that received intervention (Table 4). However, for the normal+HVPC(-) group, this difference was significant (p<0.0001) only when compared with the value obtained at 24 hours (Table 4). There was no difference in serum histamine between the three experimental groups at 48 hours (p=0.20; Table 4). However, the inflamed+HVPC(-) group presented the lowest histamine (Table 4).

**Table 4.** Comparison of histamine (nmol/L) between 24 and 48 hours within each group of mice.

Time	Normal Control	Normal + HVPC(-)	Inflamed + HVPC Sham	Inflamed+ HVPC(-)

24 h	3.09±3.31	9.25±3.01*	8.34±3.9*	5.68±2.68
48 h		6.64±2.76**	5.16±2.62	3.9±1.15
p (24 vs. 48 h)		<0.0001	0.18	0.30

Data are expressed as the mean ± SD.

* p<0.05 compared to normal control;

** p<0.01 compared to normal control. HVPC: high voltage electrical stimulation; p: statistical significance.

### Analysis of covariance

The analysis of the effect of HVPC(-) in the inflamed group on PWL revealed a positive association β=5.84 (p=0.08) compared with the normal+HVPC(-) group. This coefficient was adjusted by PWL measurements 24 hours after induction of inflammation, by histamine and albumin at 48 hours, and by the final weight of the experimental animals. The effect of HVPC(-) on histamine presented a β=1.97 (p=0.42), similar to the inflamed+HVPC Sham group (β=1.84; p=0.43). These results were adjusted by the histamine measurements 24 hours after induction of inflammation, and also by measurements of pain, inflammation, and albumin 48 hours after induction of inflammation and the final weight of the animals (Table 5).

**Table 5.** Analysis of covariance for dependent variables. Comparison group: Normal+HVPC(-).

Variable	Inflamed + HVPC Sham Placebo	Inflamed + HVPC(-)
	β (p)	β (p)
PWL time (s) 48 h	-----	5.84 (0.08)
Volume (mL) 48 h	-----	-0.35 (0.24)
Albumin (g/dL) 48 h	-0.09 (0.58)	-0.18 (0.41)
Histamine (nmol/L) 48 h	1.84 (0.43)	1.97 (0.42)

HVPC: high voltage electrical stimulation; PWL: paw withdrawal latency time. s: seconds; h: hours; mL: millilitres; ß: association; p: statistical significance; g/dL: grams/deciliters; nmol/L: nanomol/liter.

## Discussion

HVPC(-) applied at a submotor level 45 minutes per day for three consecutive days was not effective in reducing pain or edema in the joint inflammation induced by ι-carrageenan. Furthermore, analysis of covariance, adjusted by PWL measured 24 hours after induction of inflammation, by the final weight of the animal, and by albumin and histamine levels 48 hours after induction of inflammation, demonstrated a positive association (β=5.84) with borderline significance, indicating increased pain in the inflamed+HVPC(-) group. These results are exciting because several of the parameters used are similar to those used in previous studies^1^^,^ 4 ^-^ 8 ^,^ 10 ^,^ 11 ^,^ 13 ^-^ 15, which allowed for comparisons.

Carrageenan injection is an inflammatory model frequently used in animals to assess the inflammatory process and the effectiveness of drugs and physical resources in the inflammation and pain treatment^25^^,^ 26. ι-Carrageenan sensitizes the primary afferents and generates primary hyperalgesia in the injury site^27^. Next, there is a high production of nitric oxide, prostaglandins, free radicals, and cyclooxygenases that activate the dorsal horn neurons, generating a central sensitization, spinal or supraspinal, which in combination with an increased sensitivity of peripheral nociceptors is manifested as a secondary hyperalgesia^27^. Using the same experimental model, Sluka et al.^21^ and Resende et al.^26^ showed reduced primary hyperalgesia by applying high-frequency transcutaneous electrical nerve stimulation (TENS) (100 Hz and 130 Hz, respectively), similar to that used in the present study. Our results show that the inflamed+HVPC placebo group presented the lowest PWL, and the inflamed+HVPC(-) group presented longer PWL, though without a significant difference. These results indicate that HVPC(-) may increase the threshold of excitability in the primary afferent neurons and help control the pain. A possible cause of the lack of significance in the PWL values could be the short duration of the HVPC pulse, which did not allow for the stimulation of the Aδ receptors or inhibition of the medullary dorsal horn neurons^26^.

The inflammatory process induced by carrageenan observed in the present study was similar to previous studies^20^^,^ 22 ^,^ 23 ^,^ 27. These studies reported the presence of pain and edema in the first 5 hours, which persisted for up to 24 hours and then decreased. In our study, HVPC(-) did not promote greater reduction of edema in the inflamed+HVPC(-) group. Accordingly, our results are not in agreement with those obtained in previous studies that demonstrated significant differences in the reduction of trauma-induced edema in rats^3^^-^ 8 ^,^ 13. Perhaps the mechanisms of the induction of inflammation (carrageenan versus trauma) influenced these different results.

The HVPC parameters used in the present study are the same as in previous studies regarding the type of current^1^^,^ 4 ^-^ 8 ^,^ 10 ^,^ 11 ^,^ 13 ^-^ 15, level of stimulation^4^^-^ 8 ^,^ 15, polarity^1^^,^ 4 ^-^ 8 ^,^ 10 ^,^ 11 ^,^ 13 ^-^ 15, and frequency, as well as the application of the stimulation immediately after the induction of inflammation. The differences in our protocol were the mechanism of injury and the duration of the current.

Previous studies^3^^-^ 8 ^,^ 13 have used a traumatic incident to generate the inflammation, while the administration of carrageenan was the choice in the current study. Carrageenan induces inflammation and particularly produces the presence of polymorphonuclear cells in the synovial fluid during the acute phase, followed by proliferation and infiltration of the synovial membrane; the number of cells in the synovial fluid gradually decreases 24 hours after the inflammation induction^28^. This model was chosen because it produces a homogeneous pattern in the involvement of soft and joint tissues. This homogeneous pattern of inflammation between the animals cannot be guaranteed in models using mechanical trauma because the joint is injured by hitting the paw, without ensuring the generation of a controlled joint inflammation. Furthermore, the carrageenan model is a validated model for studying the inflammatory process^25^^,^ 26. Our results indicate that HVPC(-) was not effective at controlling the inflammatory process induced by carrageenan, possibly due to the involvement of the cartilage and synovial tissue, as well the parameters used, which will be discussed below. Further studies should be conducted to assess the articular cartilage and the presence of circulating inflammatory cytokines, such as IL-6 and TNF-α.

The present study used a 45-minutes session of HVPC(-) on three consecutive days in an attempt to mimic what is performed in the clinical practice. Previous studies^4^^,^ 6 ^-^ 8 that reported a decrease in edema applied three or four 30-minute applications on the same day that the lesion was established (i.e. 1.5 to 2 hours per day) with a 30-minute rest between the applications. An increase in edema was observed in the periods of non-intervention. Compared to our study, these results indicate that the duration of the application can be critical in reducing edema, as proposed by Mendel and Fish^29^, who suggested the use of longer applications during the acute stage of inflammation. Future studies in humans should assess the effect of the duration of the HVPC(-) application on acute inflammation.

Albumin was reduced in the groups submitted to inflammation at 48 hours. The acute inflammatory phase generated by carrageenan is characterized by an increase in circulating cytokines, such as IL-1, IL-6 and TNFα, which produce two effects31. The first effect is the increased microvascular permeability that allows a large loss of plasma proteins, which accumulate in the interstitial space. The second effect is a reduced synthesis and release of negative acute phase proteins, such as ferritin and albumin^28^. These factors result in hypoalbuminemia, which was observed 48 hours after induction of inflammation, especially in the animals submitted to inflammation.

On the other hand, a less strong response was found in the animals from the normal+HVPC(-) group, which can be explained by the reduction in plasma volume and anesthesia, in agreement with the study conducted by Renkin et al.^30^. Another factor that may have influenced the decrease in albumin was the weight loss observed in the animals. There was no difference in albumin level between the two inflammation groups, which is consistent with previous studies addressing paw volume and indicates no effect of HVPC(-) on the permeability of the endothelium. One possible explanation is that the short pulse duration was not able to generate plasma protein movement, as suggested by Mendel and Fish^29^.

Plasma histamine was selected due to its importance as a mediator of acute inflammation. Furthermore, the local administration of carrageenan produces a systemic reaction in response to local inflammation, which is consistent with an acute-phase response^31^. Differences were observed in serum histamine between the inflamed+HVPC placebo group and normal+HVPC(-) groups compared to the normal control group 24 hours after the beginning of the experiment; the higher increase was observed in the normal+HVPC(-) group. Two previous studies^32^^,^ 33 have reported an increase in the histamine precursor histidine decarboxylase in skeletal muscle after direct application of HVPC that peaks 8 to 12 hours after the stimulation. The increase in histamine precursor depended on the intensity and duration of the stimulation^32^. This pro-inflammatory effect may be associated with the local stress produced by the current and with the metabolic changes at the application site. Further studies are needed to confirm these hypotheses.

However, the histamine levels observed in the animals from the inflamed+HVPC group were not significantly different from the other groups 24 and 48 hours after induction of inflammation. Similarly, the analysis of covariance did not reveal significant differences (Table 5). This result indicates that during the acute phase of the inflammatory process, HVPC(-) was not able to regulate histamine release or affect the histamine binding sites in the postcapillary venule endothelial cells, which is contrary to the hypothesis proposed by Taylor et al.^3^. However, the histamine levels observed in the inflamed+HVPC(-) group 24 and 48 hours after induction of inflammation were lower than the ones observed in the inflamed+placebo group, suggesting that electrical stimulation could help control inflammation even with no change in clinical parameters, such as edema. Therefore, further studies should address more sensitive markers of inflammation, such as TNFα or IL-1.

### Clinical implications

Although the HVPC(-) was not effective, at a significance level of p<0.05, this current influenced the release of mediators such as histamine as well as the PWL in animals submitted to HVPC(-). Furthermore, HVPC(-) promoted an increase in histamine in normal animals. However, it is necessary to analyze the physiological implications of these results and also whether they also occur in humans.

## Conclusions

HVPC(-) with the parameters used in this study and applied in the acute phase of joint inflammation induced by carrageenan can regulate histamine and increase the PWL. However, it was not effective at reducing edema. Further studies are needed to validate the use of HVPC(-) in the treatment of acute inflammation.
